# Fairness in assessment practices in online education: Iranian University English teachers’ perceptions

**DOI:** 10.1186/s40468-022-00164-7

**Published:** 2022-06-01

**Authors:** Zeinab Azizi

**Affiliations:** Department of Teaching English and Linguistics, Faculty of Literature and Humanities, Ayatollah Borujerdi University, Borujerd City, Iran

**Keywords:** Fairness, Assessment practices, Online education, University English teachers

## Abstract

Although fairness in assessment practices (APs) in traditional classes has gained noticeable attention in recent years, it has remained unexplored in online education (OE). Thus, this study explores Iranian university English teachers’ perceptions of fair APs in OE. For this purpose, 21 university English teachers from Lorestan University and Ayatollah Borujerdi University, Iran, were selected using a purposive sampling method. They were invited to express their conceptions of fair APs by completing a reflective written statement questionnaire. The collected data were subjected to a thematic coding analysis. The results yielded three overarching categories: distributive justice (i.e., equality should be considered, equity is of paramount importance, and assessment practices should be tied with students’ needs), procedural justice (i.e., voices of students should be heard, both consistency and flexibility are required, and assessment procedures should be transparent), and interactional justice (i.e., interpersonal justice is crucial and informational justice should be considered). The study concludes by proposing a range of implications for different testing stakeholders.

## Introduction

It is deemed that one of the crucial components of quality education is an assessment which is administered with the aim of measuring students’ learning. As Green et al. (2007) note, assessment practices (APs) in the classroom follow two purposes: assessment of learning and assessment for learning. APs are of two broad categories in the classroom: summative and formative. In the former, the results of APs are used to make high-stake decisions (e.g., college admission). In the latter, the results of APs are used to inform teaching (Fan et al., 2020). In the literature, the leaders of the field have endeavored to outline the standards of quality APs. In an attempt, the Joint Committee on Standards for Educational Evaluation (JCSEE) (2015) provided the Classroom Assessment Standards for Pre-K-12 Teachers. Based on the empirical studies, five standards for quality APs are outlined, including reliability and validity, cultural and linguistic diversity, unbiased and fair assessment, exceptionality and special education, and reflection (Green, 2009; Hamid et al., 2019; Mazzoli Smith et al., 2018; Rasooli et al., 2019; Rezai et al., 2022).

In recent years, the social psychology theory (SPT) has been proposed to define and conceptualize fair APs in the classroom (Grace, 2017; Rasooli, et al., 2019). According to Rasooli, Zandi, and DeLuca (2019), the conceptualization of fair assessment presented by SPT rests upon three main questions: “(a) what are the antecedents of students’ un/fairness perception?, (b) how do students shape their un/fairness perception?, and (c) what psychological and social consequences proceed from students’ un/fairness perception?” (p. 702). The theoretical underpinnings of these questions are built on three main principles associated with the dimensions of social psychology of justice. They include *distributive justice*, *procedural justice*, and *interactional justice* (Resh & Sabbagh, 2016). In simple terms, as Rasooli et al. (2018) define, distributive justice is related to the fairness of outcome distributions. The procedural justice deals with the fairness of procedures for outcome distribution. The interactional justice is concerned with the fairness of the communication of information and interpersonal behavior. In general, the perceptions of testing stakeholders are shaped by these three dimensions, which, accordingly, may lead to their negative or positive behavioral and affective reactions to APs in the classroom.

The previous studies have demonstrated that fair assessment is a critical factor in the classroom. For example, Holmgren and Bolkan (2014) found that fair assessment is highly linked with students’ academic achievement, Berti et al. (2010) reported that students’ engagement was determined by the fair assessment, and Chory-Assad (2002) reported that when APs were perceived fair by students, their motivation increased significantly. In contrast, Ishak and Fin (2013) uncovered that unfair assessment was significantly correlated with the truancy of students, Murdock et al. (2007) disclosed that cheating increased when students found APs unfair, and Chory-Assad and Paulsel (2004) showed that one of the strong predictors of students’ hostility and aggression was unfair APs in the classroom. Of particular note is that the major part of the previous attempts has been allocated to conceptualizing fairness in face-to-face classes which have led to “classroometric theories” of assessment (Brookhart, 2003; Rasooli et al., 2018). Over the last years, especially with the emergence and dissemination of the COVID-19 pandemic, online education (OE) has become the primary education style for students around the world. The empirical findings have reported though OE is flexible and cost-effective and offers a wider range of learning resources, it is quite different from the face-to-face classes. It demands teachers and students to modify their ways of instructing and learning. In actual fact, teachers are obliged to accommodate novel teaching and assessment approaches such that they meet students’ needs and wants.

University teachers’ perceptions are of paramount importance to further our understanding of fairness in APs in OE. Investigation into university teachers’ perceptions of fairness in APs can be useful to promote their assessment literacy and guide them to make fair decisions about students’ abilities. Additionally, engaging university teachers in conversation about fairness in APs may raise their awareness of the issue and help them implement quality APs in OE. Furthermore, it is quite essential to take into account the distinctiveness of APs in OE. The last justification for conducting the present study is that, to the best knowledge of the researcher, the university teachers’ perceptions of fair assessment in OE have remained unexplored in Iran. Hence, the present study aims to further our understanding of the Iranian university teachers’ perceptions of fairness in APs in OE.

### Theoretical foundation

Approaching fairness from the lens of the SPT traces back to political, legal, and organizational settings (Rasooli et al., 2019). The attempt was directed to disclose how fairness is perceived by individuals in the workplace and how they react cognitively, affectively, and behaviorally to fairness (Kazemi, 2016). As noted above, the SPT approaches fairness from three different perspectives.

The first perspective is *distributive justice*. It aims to show how the outcomes of fairness are distributed (Kazemi & Törnblom, 2008). It includes three principles: equality, equity, and need. The equality principle prescribes that the outcomes should be distributed equally among students (Greenberg, 2011). The equity principle suggests that there should be a just ratio between the time and efforts students put in and the results they obtain (Murillo & Hidalgo, 2020). The need principle proposes that the outcomes should be distributed in line with students’ needs (Rasooli et al., 2019).

The second perspective is *procedural justice*. Its aim is to show if the procedures for the distributions of the outcomes are fair (Rasooli et al., 2018). It comprises diverse principles, including consistency, bias suppression, accuracy, correctability, voice, and ethicality (Rasooli et al., 2019). The consistency principle proposes that the procedures should be implemented consistently. The bias suppression prescribes that the implementation of procedures should be neutral. The accuracy principle suggests that the procedures should be administered adequately. The correctability recommends correcting the procedures if they are implemented wrongly. The voice principle suggests that students’ voices and ideas should be taken into account during the implementation of procedures. The ethicality principle is based on the premise that the implementation of procedures should be ethically aligned (Rasooli et al., 2019).

The third perspective is *interactional justice*. It refers to the social dimension of fairness (Rasooli, Zandi, & DeLuca, 2019). It entails two principles, namely, interpersonal justice, and informational justice. The interpersonal justice principle prescribes that students should be treated respectfully and politely. However, the informational justice principle suggests that students should receive truthful, adequate, and honest information (Rasooli et al., 2018).

Although a range of recent studies has explored fairness in APs in face-to-face classes (Grace, 2017; Rasooli et al., 2019, Fan et al., 2020; Murillo & Hidalgo, 2017, 2020; Resh & Sabbagh, 2016), it can be argued that more empirical studies are needed to explore university teachers’ perceptions of fairness in APs in OE. In a sense, it is essential to explore if the distributive justice, procedural justice, and interactional justice are important to consider APs fair in OE in Iranian higher education contexts.

### Teachers’ conceptions of fairness in assessment practices

As an organized system of beliefs, conceptions are shaped when an individual experiences a phenomenon and interacts with it (Coll & Remesal, 2009; Murillo & Hidalgo, 2020). As Van den Berg (2002) notes, conceptions are constructed and consolidated within the interactions of an individual with the world and they bring about a strong social quality. In relation to the field of education, teachers’ conceptions are viewed as a set of structured beliefs which are shaped due to their interactions with the classroom (Marshall & Drummond, 2006). Also, teachers’ conceptions are affected by their professional development and practices (Murillo & Hidalgo, 2020; Rezai et al., 2021). Without a doubt, teachers’ conceptions play a crucial role in the instructional and assessment processes in the classroom (Brown & Gao, 2015).

In the literature, teachers’ conceptions have gained noticeable attention in the studies conducted by Brown (2003, 2004, 2006). Brown and colleagues have conducted a range of studies to disclose teachers’ and students’ conceptions of fair assessment to determine their implications for the classroom. They found that teachers’ conceptions of fair assessment revolved around four key points: (a) the accountability of schools is linked with APs, as they determine the efficacy of schools’ operations; (b) the accountability of students is correlated with APs, as they measure their performance; (c) education is improved due to the positive effects of APs; and (d) APs are irrelevant when students perceive them unfair. Additionally, Tierney et al. (2011) carried out a study on teachers’ perceptions of fair assessment in Canada. They came up with a number of key points: “teachers assessed students on what they believed was right for them, for the individual good of each student and the common good of the classroom and school” (p. 21). Likewise, in a multi-case study, Tierney (2014) attempted to explore primary and secondary teachers’ conceptions in Canada to re-conceptualize equitable fair assessment. The findings evidenced that in order for APs to be considered fair they should incorporate multiple learning opportunities, be transparent, lead to creating a trustful climate in the classroom, lead to promoting critical reflection, and it should not lead to equal evaluation. Further, Murillo and Hidalgo (2020) carried out a phenomenographic study to disclose the Spanish teachers’ conceptions of fair assessment in the classroom. Their findings documented that the participants’ conceptions of fair assessment revolved around the equality and equity principles. Additionally, they found that the participants’ conceptions were affected by the school context. As can be implied from the reviewed studies, fair assessment in OE has been overlooked, a gap that the present study aims to fill in.

### Assessment practices in online education

With the development of modern technologies and learning tools and systems, OE has been receiving ongoing attention from day to day. Therefore, APs should be accommodated in such ways that they can measure students’ learning accurately and adequately (García-Peñalvo et al., 2021; Sa’di et al., 2021). APs in OE which have been termed e-assessment can bring about both advantages and disadvantages (St-Onge et al., 2021). The advantages include time and location flexibility, lesser administrative burden, easier preparation, scoring and moderating of question papers, quicker evaluations and results, a friendly climate, a secure solution, easier report creation, and cost-effectiveness (St-Onge et al., 2021; Kundu & Bej, 2021). However, the disadvantages include challenges in technology adoption, infrastructural barriers, difficulty in grading long-answer type, susceptible to cheating, transitioning to open-book exams, and the lack of face-to-face interactions between teachers and students (García-Peñalvo et al., 2021; Kundu & Bej, 2021; Sa’di et al., 2021). For example, in e-assessment practices, students can answer on their own devices at home; thus, they cannot be checked upon. As the second drawback, students do not have opportunities for raising their concerns and sharing their voices about e-assessment practices.

Taken together, it is reasonable to argue that fairness is a critical facet in e-assessment practices. Therefore, the conceptualization of fairness should be reshaped in e-assessment practices such that it can be useful for testing stakeholders to implement quality e-assessment procedures, leading to quality education. For this purpose, the present study purports to create an initial empirical foundation to re-conceptualize fairness in e-assessment practices built on Iranian university teachers’ perceptions.

## Method of the study

### Research design

The researcher used a ground theory design to conduct the present study. As Cresswell and Poth (2018) note, it is a qualitative method used by researchers to survey a particular phenomenon to discover new theories based on real data. Hence, to further our understanding of the ways through which university teachers perceived APs as fair, this study used a grounded theory design.

### Setting and participants

This study was conducted in the settings of Lorestan University and Ayatollah Borujerdi University in Lorestan Province, Iran. Using a purposive sampling method, the researchers selected 21 university teachers who were working at the Department of Teaching English at the time of conducting this study. According to Riazi (2016), as a non-probability sampling, researchers use purposive sampling to choose individuals in a population based on their own judgment. The researcher selected the participants in terms of major, gender, teaching experiences, and academic rank to satisfy the theoretical sensitivity. The participants’ demographic information is reported in Table 1.

**Table 1:** The participants’ demographic information

Participant	Gender	Rank	Major	Teaching experience
Nazanin	F	Asso Pro.	Applied Linguistics	15
Reza	M	Assis Pro.	Linguistics	12
Mohsen	M	Asso Pro.	Translation	24
Zivar	F	Assis Pro.	English literature	8
Farshad	M	Visiting Lect	Applied linguistics	5
Ramin	M	Asso Pro.	Applied linguistics	12
Mona	F	Assis Pro.	Applied linguistics	21
Hossein	M	Assid Pro.	Linguistics	18
Leila	F	Visiting Lctur.	Linguistics	9
Akbar	M	Assis Pro.	English literature	14
Azam	F	Asso Pro.	Applied Linguistics	12
Bahar	F	Assis Pro.	English literature	8
Fardin	M	Assis Pro.	Linguistics	21
Alireza	M	Visiting Lectu	Applied linguistics	3

The researcher referred to the faculty of Foreign Languages and Humanities of Lorestan University and Ayatollah Borujerdi University and submitted her proposal for research quality and ethical adherence to the Deputy and Education. She received clearance from them to pursue participants’ recruitment. As this study was conducted during the COVID-19 pandemic and university teachers were not available at the campus, the researchers took their phone numbers and contacted them. She introduced herself, gave information about the study, and asked if they were willing to participate in the current study. Afterward, she sent digital written consent to them so as to be signed and sent back to the researcher. She announced to them that their participation in this study was voluntary, and they could stop their cooperation as they wished. Of particular note is that the researcher ensured the participants that their responses would be kept confidential and they would be informed about the final results.

### Instruments and data collection procedures

The researcher used a reflective written statement to gather the required data. As Moustakas (1994) notes, researchers use a reflective written statement to encourage participants to reflect on their perceptions of a particular phenomenon. In the literature, other researchers (Gao et al., 2021; Horan et al., 2010) used a reflective statement to get participants to reflect on their perceptions of fair assessment. For this purpose, the participants were asked to reflect on the following prompt:

Dear professor,

As you know, fairness is one of the bedrocks of assessment practices in the classroom. I kindly invite you to reflect on your perceptions of the features making assessment practices perceived as fair. In actual fact, you are supposed to write down your views about the fundamental features of fair assessment practices. A report of 300–500 words in length will be sufficient.

It is worth noting that the researcher invited two university professors to read the prompt and to assess if it was fitting in terms of readability and content. Based on their comments, they modified some parts in terms of language and content. Afterward, she sent a digital format of the written reflective statement to the participants via email and WhatsApp. The participants were asked to contact the researcher if they faced any problems during the completion of the written reflective statement. The participants’ responses were stored in a database to be analyzed meticulously later. Of particular note is that she recruited a well-experienced translator to translate the participants’ words into English. The participants were allowed to reflect on their perceptions of fairness APs in Persian such that they could express their perceptions with ease.

### Data analysis procedures

The researcher used a thematic coding analysis to analyze the collected data. According to Riazi (2016), thematic coding analysis is an iterative process to extract the prominent themes from collected data. It included a six-step process. The first step was familiarization in which the researchers read the participants’ responses as much as they could understand them. She went through the data and started underlining the prominent concepts. The second step was assigning preliminary codes to the collected data to describe the content. During this step, the researcher used different colors and numbers to determine the emerging codes. The third step was searching for prominent themes. In this step, the researcher went through the extracted codes over and over to verify the major themes. In the fourth theme, the researcher reviewed the themes to make sure that they represented the intended meanings of the participants. In the fifth theme, the researcher defined and labeled themes. She tried to label the themes such that they stand for the content. The last step was producing a model where the researcher tried to present a model based on the extracted themes. Of particular note is that the researcher measured the reliability and credibility of the findings. For the former, she recruited two coding analysts to analyze the collected data independently. The results of their inter-rater reliability through Cronbach alpha yielded 0.87 which was found acceptable for the present study. Concerning the credibility, she used a member checking strategy. In doing so, she invited five participants and gave a copy of the extracted themes and excerpts to them. They confirmed that the extracted themes and excerpts were in conformity with their intended meanings.

## Results and discussion

**Fig. 1:**
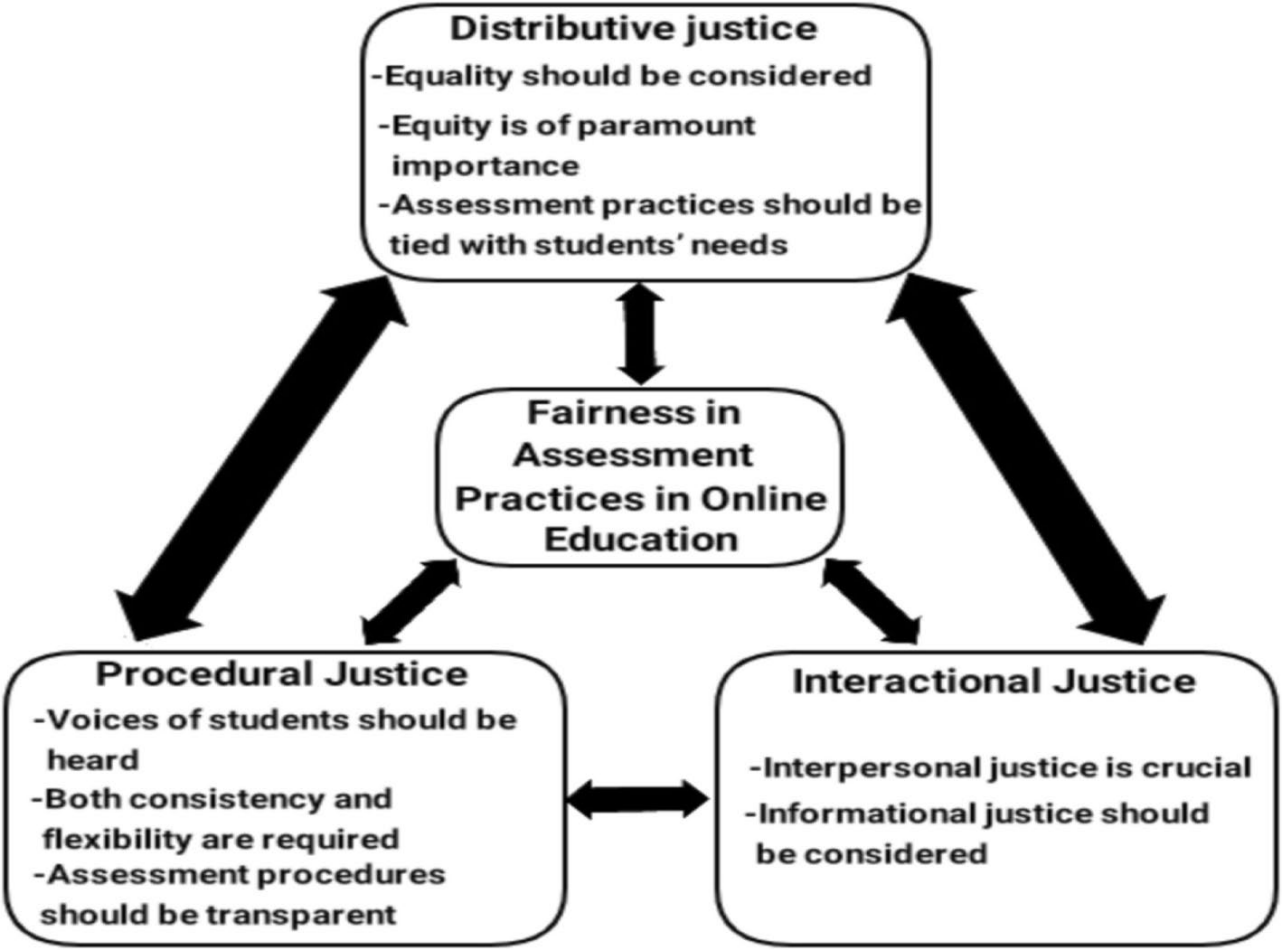
A model of fairness in assessment practices in online education

The results of the thematic coding analysis yielded three overarching categories: distributive justice (i.e., equality should be considered, equity is of paramount importance, and assessment practices should be tied with students’ needs), procedural justice (i.e., voices of students should be heard, both consistency and flexibility are required, and assessment procedures should be transparent), and interactional justice (i.e., interpersonal justice is crucial and informational justice should be considered) (Fig. 1). They are detailed below.

### Distributive justice

#### Equality should be considered

The first theme that emerged from the collected data was “equality should be considered.” The university teachers stressed that the test outcomes should be equally distributed among students. In this regard, Reza stated:

*“Assessment practices are not perceived as fair by students when the outcomes are not distributed equally among them. For example, if some students do not perform well in online assessment practices, the decision made based on the test results should be equal for all the equal. To make this important objective realized, I try to make my assessment practices as valid and reliable as possible.”*

Further, the participants emphasized that test outcomes of APs in OE should be designed, administered, and scored in line with students’ digital literacy and technological devices. In support of this, Mohsen remarked:

*“One of the crucial factors affecting students’ learning and their capabilities to show their learning is students’ digital literacy. The students who enjoy a high level of digital literacy can demonstrate their abilities better on online tests. Therefore, I consider this point when making a decision about students’ academic destiny.”*

As can be implied from the statements above, equality is a crucial dimension of distributive justice. The findings documented that concerning the distribution of the outcome, when students are treated equally, they might perceive APs as fair in OE. Along with Murillo and Hidalgo (2020), it may be argued that APs are not just if during their administration, the equality of conditions, such as resources, time, space, and materials, is not ensured. This argument receives support from previous scholars (Tierney, 2014, 2016; Camilli, 2013; Nisbet, 2019; Rasooli et al., 2018; Rasooli, et al., 2019; Rasooli, Zandi, & DeLuca, 2019), arguing that one of the fundamental premises for fairness in APs is impartiality. And, along with Worrell (2016), the findings revealed that to achieve this valuable purpose, university teachers should measure students’ learning adequately through reliable and valid APs in OE. Hence, equality is a pillar of distributive justice in APs in OE.

#### Equity is of paramount importance

The second theme germane to the distributional justice was “equity is of paramount importance.” The university teachers were of the opinion that APs in OE should lead to establishing a balance between students’ time and effort and the obtained results. For this, Zivar remarked:

*“Assessment practices should be designed, administered, and graded in such a way that students feel they can get the desired results. For example, assessment practices should not be formed in such a way that the students who cannot work with computers and digital devices well fail to show their abilities efficiently. For example, with the development of online classes, some students have been obliged to join them without sufficient digital literacy.”*

Additionally, the university teachers stressed that the lack of face-to-face interactions has jeopardized students’ opportunities for reaching their desired results. In this respect, Ramin quoted:

*“Due to the absence of face-to-face interactions in assessment practices, students cannot communicate their problems freely. They may lose some questions owing to the limited access to teachers to remove the possible ambiguity. Thus, they cannot reach their favorable scores.”*

As the excerpts above revealed, one of the critical dimensions of distributive justice is equity. The findings indicated that when students find the ratio of their allocated time and energy with the obtained scores, as well as the ratio of their scores with other peers proportionate, APs are perceived as fair (Rasooli, Zandi, & DeLuca, 2019). In a sense, based on the findings, it may be argued that when a student’s contribution-to-outcome ratio is equal to other students’ contribution-to-outcome ratios, they might perceive APs as fair in OE. In contrast, when students observe the distribution is not proportional, they might reach the injustice perception (Murillo & Hidalgo, 2017). The findings of the study received support from the previous studies (Rasooli et al., 2018; Murillo & Hidalgo, 2020; Tierney, 2014), reporting that teachers considered APs as unfair if the relation between input and outcome was disproportional. In short, the equity principle is vital in APs in OE.

#### Assessment practices should be tied with students’ needs

The third theme linked with the distribution justice was that “assessment practices should be tied with students’ needs.” The university teachers expressed that the outcomes of APs should be distributed in line with the needs of students. To support this, Akbar commented:

*“Assessment practices are not fair unless they are designed according to students’ needs in online classes. When university teachers encourage students to participate in assessment practices, for example, by implementing alternative assessment practices like peer-assessment and self-assessment, they can meet their needs well.”*

Additionally, the participants stressed that APs that are adapted to students’ characteristics and needs are considered fair. In this regard, Mona pinpointed:

*“As students learn differently, they tend to show their abilities differently too. This is more tangible in online classes where the assessment practices need to be designed and administered in different ways. For example, some students prefer to respond to closed-ended questions while other students like to answer open-ended questions.”*

As the statements above indicated, one of the key principles of distributive justice is the needs of students. The findings evidenced that APs should be designed, administered, and graded in line with students’ needs. As Tierney (2016) argues, when the distribution of assessment processes is carried out in line with students’ needs, it is perceived as fair. Along with Rasooli, Zandi, and DeLuca (2019), it may be argued that APs in OE should be adapted to the needs and lacks of students. One of the strategies that may make the way for this is increasing students’ participation in APs (Picón-Jácome, 2013). For example, as the results showed, teachers can take into account student diversity by administering alternative and multiple APs with different instruments.

### Procedural justice

#### Voices of students should be heard

The first theme connected with procedural justice was “voices of students should be heard.” The participants posited that students should be given an opportunity to cooperate in designing, administering, and grading in APs. For this, Alireza said:

*“I believe that tests are to improve students’ learning. Therefore, I try to consider my students’ opinions and views. For example, last semester, my students could not get good marks on the final test because they did not know how to take the online test. They appealed for re-administering the test. At the second time, I observed that they demonstrated their abilities better and felt satisfied with the test.”*

Another important point verified by the participants was sharing students in decision-making processes. They commented that when students are engaged with decision-making processes, they perceive APs as fair. In this respect, Farshad underlined:

*“The decisions made based on test results in online classes should be in cooperation with students. For example, in line with students’ opinions, I lowered the cut-score to 8. The reason is that my students were blamed for the additional workload in the online courses. They complain that vis-à-vis traditional classes, they have to put more time and energy into studying materials and doing the assignments. I found their explanations persuasive and lowered the cut-score.”*

As can be inferred from the statements above, university teachers should consider the voices of students in APs. The results evidenced that students should have the opportunity to express their concerns and ideas about APs in OE. According to the findings, it may be argued that when university teachers do not open windows for students to articulate their voices about assessment procedures, it may exert negative effects on students’ perceptions of fairness (Murillo & Hidalgo, 2017). Along with Tata (2005), the findings demonstrated that university teachers should allow students to participate in assessment procedures such that they feel ownership over grading criteria, workload, punishment, learning materials, missed work, and make-up classes. The study’s findings are in line with Schmidt et al. (2003), reporting that when students were provided with the opportunity for appealing for their grades, they were more likely to conceive APs fair. To close, students should not be deprived of voices concerning the assessment procedures.

#### Both consistency and flexibility are required

The second theme germane to procedural just was “both consistency and flexibility are required.” Though consistency and flexibility seem contradictory, the university teachers stressed that they are both required to implement fair APs in OE. Concerning the importance of consistency, Bahar remarked:

*“The assessment practices should be administered consistently for all students. I mean that the content of tests, the types of the tests, and the time should the same for all students. For example, it is not fair if I give close-ended tests to a part of the students and, concurrently, give the open-ended tests to the other part of the students. The students should not feel distinguished.”*

Simultaneously, the participants emphasized that APs sometimes should be designed and administered flexibly. In this respect, Azam opined:

*“Opposed to face-to-face classes, the conditions in online classes are totally different. You know that holding online classes is heavily dependent on the Internet connectivity issues. When I am going to administer an online test, unfortunately, my students lose their internet connectivity. I have to give more time to them or even I have to design and administer the test once more. Otherwise, students cannot show their abilities accurately.”*

The university teachers’ words clearly indicated that assessment procedures should be both consistent and flexible. The results disclosed that university teachers should apply assessment procedures consistently from designing to making decisions. Aligned with the previous studies (Camilli, 2006; Horan et al., 2010; Leventhal, 1980; Rasooli, Zandi, & DeLuca, 2019; Robbins & Jeffords, 2009; Rodabaugh, 1994), the findings indicated that there should be consistency in promise-keeping, course content, attendance policy, punishment, and grading*.* The worthy point to note is that though the participants perceive the consistent implementation of assessment procedures as fair, they underlined the importance of flexibility to accommodate the particular conditions of students. The results are in congruent with those of Robbins and Jeffords (2009), reporting the significance of consistency. Additionally, the findings lend credence to the results of Whalen and Koernig (2009). They reported that students longed for teachers’ flexibility in accommodating their special conditions. In short, consistency and flexibility should be accommodated in assessment procedures in OE.

#### Assessment procedures should be transparent

The third dominant theme related to procedural justice was “assessment procedures should be transparent.” The university teachers highlighted that the information about APs should be accurate, transparent, and explicit. In support of this, Liela commented:

*“If the aim is to help students to perceive assessment practices as fair, university teachers should provide students with explicit information about assessment practices. I mean they should clarify the contents of tests, the kinds of items, the procedures of test administrations, the scoring criteria, and the decisions that will be made based on test results. All things should be clear for students where teachers can share this information via voice podcasts, for example.”*

Further, the participants underscored that as assignments are an integral part to assess students fairly, students should be notified about their requirements in OE. Hossein put it in this way:

*“In online education, it is necessary to provide a situation in which students know everything about assignments. It makes them perceive the final scores as fair. For this, for example, I usually establish a WhatsApp group at the beginning of the course. Through it, I explain clearly the assignments and their importance in the final evaluation. During the course, the students send their assignments in it. I check them and offer feedback on them. I feel that this has made my assessment to be conceived fair by students.”*

The quotations above evidenced that assessment procedures in OE should be transparent for students. The findings, in sense, documented that there should be a clear enactment of assessment procedures. Along with Tierney (2014), the findings indicated that if there is a lack of transparency in assessment procedures, they may be perceived as unfair. It can be argued that if the information about assessment procedures is not transparent, teachers’ decisions for students may not be perceived as fair (Grace, 2017). The important point to note is that transparency should be invoked in all the stages of assessment procedures, such as attendance policy, course materials, grading criteria, and accommodation (Pepper & Pathak, 2008). The results of the study are in agreement with those of Pepper and Pathak (2008), reporting that students considered the explicit grading description as fair compared to the students who did not receive any information about the grading criteria. Further, the study’s findings are in congruent with those of Duplaga and Astani (2010). They found that for meeting fair criteria, students should be notified earlier about the homework collection schedule.

### Interactional justice

#### Interpersonal justice is crucial

The second frequent theme related to interaction justice was “interpersonal justice is crucial.” The university teachers underlined that the teacher-student relationship should be respectful and caring. In this regard, Reza remarked:

*“As students do not have access to students in online classes, university teachers should create a friendly relationship such that students can raise their questions and concerns about assessment practices. University teachers should respect their students. I mean that university teachers should be online available before, during, and after test administrations.”*

Resonating with the precedent statement, the participants stress that the power should be distributed equally between university teachers and students. Nazanin’s excerpts below show this clearly:

*“In online classes, the power should not be coercive. I mean that students should not be punished if they do not obey teachers’ words and rules. I feel that this view should not be dominated in online classes that since teachers are the most knowledgeable in the classes, all students have to obey him. Moreover, it is not fair to establish this view in online classes that the right of deciding and implementing of decisions is for university teachers because they are the authority of classes.”*

As can be implied from the above statements, interpersonal justice is crucial for fair assessment in OE. The findings uncovered that the relationship between teacher-student during APs should be respectful. This respectful relationship accommodates both verbal and nonverbal interactions. The findings are in agreement with those of Kerssen-Griep and Witt (2012) who reported that both verbal and non-verbal interactions were associated with students’ perceptions of fairness in APs. The other important point disclosed in the findings was that the university teachers’ use of power plays a key role in teachers’ perceptions of fairness. According to the findings, it may be argued that university teachers’ use of power should not be coercive. That is, if students disobey teachers, they should not be punished. Additionally, the findings indicated university teachers should not use their power in assessment procedures as experts. That is, university teachers should not impose their power because they are the most knowledgeable individual in the classroom (Rasooli et al., 2018). Moreover, the results disclosed that the use of power in assessment procedures by university teachers should be legitimate. It means that because teachers are the authority in the classroom, they should not catch the whole right and power to decide and implement all decisions. The study’s findings are in consistent with those of Paulsel et al. (2005), reporting that the distribution of power should be balanced between teachers and students in APs.

#### Informational justice should be considered

The second recurring theme related to interactional justice was “informational justice is important.” The participants pinpointed that university teachers should present the information about APs in an adequate and truthful way such that students become persuasive. In this respect, Fardin quoted:

*“In online assessment practices, it is essential to provide students with adequate information at the beginning of the course. Since students do not have easy accessibility to teachers to put forward their questions, they should know everything about assessment practices from the designing to the grading procedures.”*

Additionally, the university teachers highlighted that as learning in OE may be quite different from the traditional classes, university teachers should justify students to know how to answer the questions. In support of this, Alireza commented:

*“Well, as learning in online classes are not the same as the face-to-face classes, students are obliged to demonstrate their learning differently. For example, they have to enter LMS to find questions, write down completely their answers, and submit them. Since many of the students do not have the required digital literacy, they have difficulty answering the questions. Hence, it is up to teachers to inform and instruct students such that they can perform well with digital tests.”*

As may be inferred from the participants’ words, information justice is important. The findings indicated that the information about APs should be truthful, adequate, and persuasive. Align with Rasooli, Zandi, and DeLuca (2019), the truthfulness, adequacy, and justification of information should be invoked proactively and reactively. That is, the required information about assessment procedures should be given to students prior to, during, and after test administrations. Along with Kazemi (2016), it may be discussed that when truthful, adequate, and justified information is provided for students at all different stages of APs, it can contribute to students’ perceptions of fairness independently from other dimensions. The study’s findings are in line with those of the previous studies (Oppenheimer, 1989; Schmidt et al., 2003), reporting that the students who were justified about the grading decisions with their teachers’ truthful and adequate information found them fair. Moreover, the results of the study are in agreement with those of Buttner (2004), revealing that the lack of dishonesty, lack of attention to students’ concerns and problems, and refusing to offer correct information about assessment procedures led to students’ perception of unfairness.

## Conclusion and implications

The present study purported to disclose the Iranian university English teachers’ perceptions of fairness in APs in OE. The study leveraged the qualitative data from 21 university English teachers’ perceptions to further our understanding of fair assessment in OE. The findings yielded three overarching patterns, including distributive justice (i.e., equality should be considered, equity is of paramount importance, and assessment practices should be tied with students’ needs), procedural justice (i.e., voices of students should be heard, both consistency and flexibility are required, and assessment procedures should be transparent), and interactional justice (i.e., interpersonal justice is crucial and informational justice should be considered). The findings evidenced that similar to APs administered in traditional classes, fairness is at the heart of APs in OE. Based on the findings of this study, it can be concluded that the distribution of outcomes should acceptable, students should be treated respectfully and caringly and they should be provided with truthful, adequate, and justified information during APs in OE. To close, it is important to design, administer, and grade APs in such a way that students perceive them as fair.

The study’s findings may offer some novel contributions. Firstly, this is the first study which used a grounded theory design to further our understanding of university English teachers’ conceptions of fair assessment in OE the Iranian EFL context. Secondly, as no study has addressed the issue of fair assessment in OE from empirical perspectives, this study could offer valuable insights into improving university English teachers’ assessment literacy regarding the fairness issue. Thirdly, the findings of this study recommend university officials holding pre-service and in-service teacher training courses to make university English teachers familiar with the tenets of fairness in APs. During these training courses, attendants are supposed to increase their knowledge and skills to administer fair APs in OE. Fourthly, the results of the study suggest that university officials run some digital literacy courses for students to help them learn better in OE and, accordingly, demonstrate their learning and abilities better. Fifthly, the findings of the present study advise university English teachers to read about the features of fair APs. This may be helpful for them to be more aware of the decisions they make based on test results and work toward fair APs. This, in turn, may directly affect their teaching practices. Finally, the results of the study recommend university English teachers using the findings of the scientific studies to administer APs. For example, they need to incorporate the voices of students in APs in OE.

Considering the limitations imposed on this study, a range of suggestions for further research is presented. First, as the present study was limited to two state universities in Iran, more studies are needed to be conducted in other parts of the country to increase the external credibility of the findings. Second, since this study was carried out in the Iranian context, more research on fair assessment in OE across cultures is needed to disclose how university English teachers perceive it to reach a conclusive framework. Third, since the participants of the present study included university English teachers, interested researchers can gather qualitative data from university students to reveal how they perceive APs as fair. Last but not least, because the data were gathered through a reflective written statement, future studies can triangulate the findings by collecting data using other data collection instruments, such as semi-structured interviews, scenarios, and observations. In this way, they may provide broader and deeper insights into university English teachers’ perceptions of fairness in APs in OE.

## Data Availability

The datasets used and/or analyzed during the current study are available from the corresponding author on reasonable request.

## Abbreviations

APs: Assessment practices
OE: Online education
